# First report of an exophilic *Anopheles arabiensis* population in Bissau City, Guinea-Bissau: recent introduction or sampling bias?

**DOI:** 10.1186/1475-2875-13-423

**Published:** 2014-11-04

**Authors:** Vasco Gordicho, José L Vicente, Carla A Sousa, Beniamino Caputo, Marco Pombi, João Dinis, Gonçalo Seixas, Katinka Palsson, David Weetman, Amabélia Rodrigues, Alessandra della Torre, João Pinto

**Affiliations:** UEI Parasitologia Médica, Instituto de Higiene e Medicina Tropical, Universidade Nova de Lisboa, Rua da Junqueira 100, 1349-008 Lisbon, Portugal; Centro de Malária e outras Doenças Tropicais Instituto de Higiene e Medicina Tropical, Universidade Nova de Lisboa, Rua da Junqueira 100, 1349-008 Lisbon, Portugal; Istituto Pasteur-Fondazione Cenci-Bolognetti, Dipartimento di Sanità Pubblica e Malattie Infettive, Università di Roma “La Sapienza”, Piazzale Aldo Moro 5, 00185 Rome, Italy; Avenida Combatentes da Liberdade da Pátria, Instituto Nacional de Saúde Pública,, Apartado 861, 1004 Bissau Codex, Guinea Bissau; Royal Institute of Technology, SE-100 44 Stockholm, Sweden; Department of Vector Biology, Liverpool School of Tropical Medicine, Liverpool, L3 5QA UK

## Abstract

**Background:**

The malaria vector *Anopheles arabiensis* exhibits greater behavioural and ecological plasticity than the other major vectors of the *Anopheles gambiae* complex, which presents challenges for major control methods. This study reports for the first time the presence of *An. arabiensis* in Antula, a suburb of Bissau city, the capital of Guinea Bissau, where high levels of hybridization between *Anopheles coluzzii* and *An. gambiae* have been reported. Given that previous surveys in the area, based on indoor collections, did not sample *An. arabiensis*, the possibility of a recently introduced exophilic population was investigated.

**Methods:**

Larval and adult mosquito collections were carried out in Antula at the end of the rainy season of 2010. *Anopheles gambiae* species composition, determined by rDNA-IGS and SINE200X6.1 markers, was compared with four previously collected samples dating back to 1993. Analysis of ten microsatellites was used to estimate levels of genetic diversity, relatedness and to investigate demographic stability.

**Results:**

*Anopheles arabiensis* comprised 54.0% of larvae and 25.6% of adults collected in 2010, but was absent in all previous collections, a highly unlikely observation by chance if the population was stable. This species had the lowest levels of genetic diversity, highest relatedness and, along with *An. gambiae*, exhibited evidence of a recent population expansion.

**Conclusions:**

Results point to the presence of a previously undetected outdoor population of *An. arabiensis* in Antula, which appears to have expanded recently, highlighting the importance of complementing indoor-based mosquito collections with sampling methods targeting outdoor adults and immature stages for a more complete assessment of mosquito biodiversity. A change in temporal dynamics in the species complex composition was also detected. Coupled with previous evidence of asymmetric introgression from *An. coluzzii* to *An. gambiae*, this suggests that the study area may be subject to ecological changes with a potential impact on both the genetics of these species and on malaria transmission.

**Electronic supplementary material:**

The online version of this article (doi:10.1186/1475-2875-13-423) contains supplementary material, which is available to authorized users.

## Background

For more than a century some of the sibling species of the *Anopheles gambiae* complex have been recognized as the most important Afrotropical malaria vectors, responsible for hundreds of thousands of deaths each year [1]. They are *Anopheles gambiae* and *Anopheles arabiensis*, which share a very widespread sympatric range. Of these two species, *An. gambiae* is considered a more efficient malaria vector due to its higher anthropophily.

A process of ecological speciation occurred within *An. gambiae* leading to the differentiation of two taxonomic units, preliminarily named M and S molecular forms [2] and lately elevated to species status, with the M-form being named as *Anopheles coluzzii* and the S-form keeping the original designation *An. gambiae* [3]. The two species co-exist in sympatry in West and Central Africa and hybrids between them are rare throughout most of their distribution range [4], although assortative mating may periodically or locally break down [5–9]. *Anopheles coluzzii* and *An. gambiae* differ in a few bio-ecological features (see [10] for a review). One of the most notable differences is the greater propensity of *An. coluzzii* to exploit larval habitats of more permanent nature (e.g., rice fields) due to superior predator avoidance when compared to *An. gambiae*, which predominates in temporary larval habitats [11–13]. Both types of larval habitats may also be colonized by *An. arabiensis* although recent evidence suggests that this species shares similar ecological requirements with *An. gambiae* in exploiting temporary larval habitats [13].

Guinea Bissau has recently become a focus of interest for studies aimed at better understanding the process of ecological speciation which led to the differentiation of *An. coluzzii* and *An. gambiae*, where exceptional hybrid rates up to *ca*. 25% have led to the hypothesis that the country may contain the core of a secondary contact region between the two species [5, 7, 8, 14, 15]. Deeper studies in this geographical region are expected to help clarify the reproductive isolating mechanisms between the two species, as well as to identify possible ecological determinants of their breakdown.

Previous reports on the *An. gambiae* complex species distribution in Guinea Bissau [16–19] show that *An. gambiae* was widespread throughout the country, while *An. arabiensis* was only present in the northern inland region, where drier open savannah and shrubland landscapes prevail. In addition, *Anopheles melas* was reported in the coastal region, characterized by flooded areas of mangrove and forest, as expected, based on its specific adaptation to brackish water larval habitats. These studies were mostly based on indoor-resting mosquito collections by pyrethrum spray catches or hand aspirations and, thus, only provide a picture of the endophagic and endophilic fraction of the local anopheline species composition in the region. In this context, larval collections may deliver a more unbiased sampling, irrespective of the feeding or resting patterns [20]. Moreover, the older studies predate the description of *An. gambiae* molecular forms. Nevertheless, the sympatric presence of *An. coluzzii*, *An. gambiae* and *An. melas* in the coastal area, where the capital city Bissau is located, has been shown in mosquito samples collected in 1995 and 1996 [21] that were subsequently identified to molecular form by Oliveira *et al*. [5].

In this study, *An. gambiae* complex species composition was assessed in Antula, a suburb of the capital city Bissau, in indoor-collected adult samples as well as in larval samples. The primary objective was to assess larval spatial segregation possibly associated to niche partitioning between *An. coluzzii* and *An. gambiae* in this secondary contact zone. In the course of the analysis *An. arabiensis* was identified for the first time in the area, which predominated in larval samples. Microsatellite data was used to determine whether the presence of this vector could be a recent introduction or a well-established exophilic population that had not been sampled earlier in the area, owing to dependence on indoor adult collections.

## Methods

### Mosquito collections

The study took place in Antula (11°50’N, 15°30’W), a semirural suburb surrounded by flooded plains, mangrove swamps and subsistence agriculture plots, located about 5 km north of the centre of Bissau, Guinea Bissau’s capital city [22]. The suburb is bordered eastwards by a large rice field. The majority of houses are clay-walled and thatch-roofed dwellings. Domestic animals (pigs, goats, chicken, and cattle) are frequent and are sometimes kept inside houses [5, 22]. The use of bed nets for protection against malaria transmission has been implemented in the study site at least since 1995 [21] and insecticide-treated nets have been introduced in the area of Bissau since 2005 [23, 24]. The climate of this region is tropical humid, with a rainy season from May to October and a dry season from November to April. Mosquito sampling was carried out at the end of the rainy season, between 8 and 28 October, 2010.

**Figure 1:**
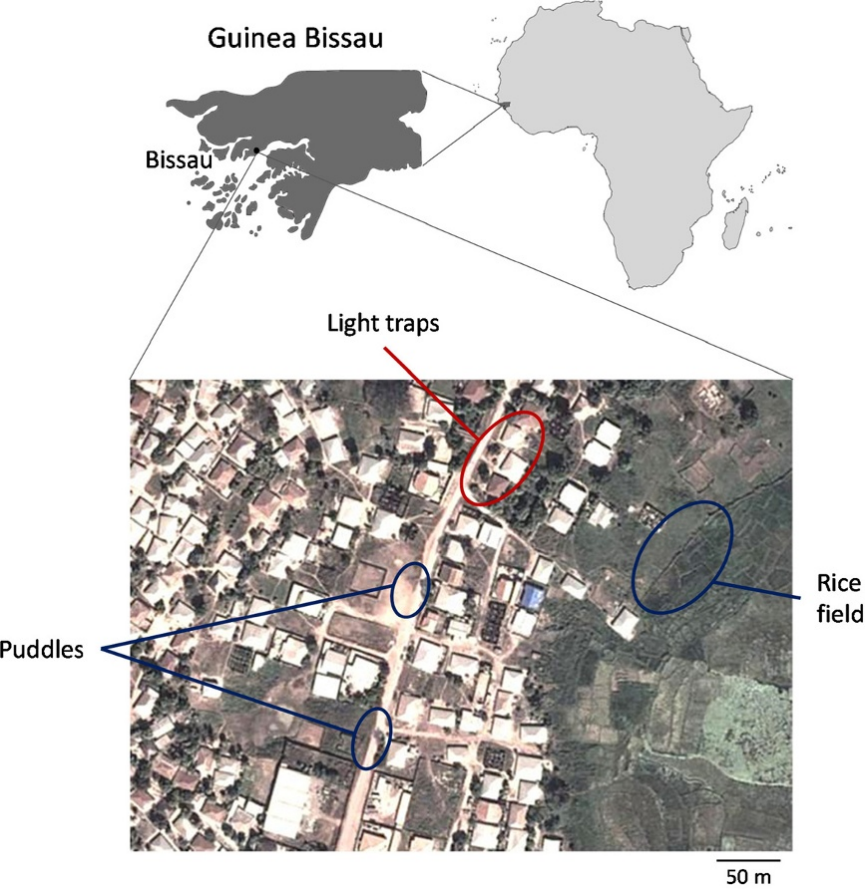
**Map of Antula**, **Bissau** **(Guinea Bissau)** **showing the collection sites for larvae and adults** **(adapted from Google Maps).**

Mosquito larval collections were performed using dips and pipettes from permanent and temporary larval habitats (Figure 1). The temporary larval habitats consisted of two main rain-water puddles bordered by smaller pools of various origins, such as tyre tracks or footprints, located on a dirt road. The permanent larval habitat was the rice field located to the east of the suburb. Larval samples were identified to the subfamily and anopheline mosquitoes were kept individually in 0.5 ml tubes filled with 80% ethanol. Adult mosquitoes were collected indoors by two methods: i) CDC light traps [25]; and, ii) resting collections, either with mechanical aspirators or by pyrethrum spray catches. Adults were identified to species or species complex with the help of digital keys [26] and kept individually in silica gel filled 0.5 ml tubes.

In addition to 2010 collections, samples of indoor-resting *An. gambiae* s.l. females collected in the same study site, *i.e*. Antula, in September/October 1993, October 1995, November 1996, and August/September 2007 were also analysed for a temporal comparison of the *An. gambiae* complex species composition in the area. Further details on these samples are described elsewhere [5, 21, 27].

### Molecular analyses

DNA extraction of larval samples was performed with the DNeasy® Blood & Tissue Kit (QIAGEN, Hilden, Germany) whereas the protocol described in Collins *et al*. [28] was used for adult mosquitoes.

*Anopheles gambiae s.l*. samples were identified to species by two complementary methods: i) the PCR-RFLP protocol of Fanello *et al*. [29] which targets species-specific polymorphisms at the intergenic spacer region of the ribosomal DNA (hereafter termed IGS); and, ii) a PCR assay targeting the insertion of the short interspersed element SINE200X6.1 (hereafter termed SINE) found to be fixed in *An. coluzzii* but absent in *An. gambiae s.s*. [30]. Specimens were identified as either *An. coluzzii* or *An. gambiae* if they had coincident species-specific patterns for both markers. Specimens exhibiting either a consistent *An. coluzzii*/*An. gambiae* pattern for both IGS and SINE or a discordant result between markers were considered as individuals of admixed ancestry [7, 31].

A subsample of larvae was sequenced for a 658 bp fragment of the cytochrome oxidase I (COI) mitochondrial gene used in mosquito DNA barcoding. Amplification by PCR was performed with the universal primers HC02198 (TAAACTTCAGGGTGACCAAAAAATCA) and LCO1490 (GGTCAACAAATCATAAAGATATTGG) [32] under the conditions described by Herbert *et al*. [33]. Amplified products were cleaned with Qiaquick® PCR purification kit (Qiagen, Hilden, Germany) and sequenced in a DNA sequencing facility (STAB VIDA, Oeiras, Portugal). Forward and reverse sequences were aligned and corrected by hand using BIOEDIT v. 7.0.5.2 [34]. Species identification was performed using the barcoding identification engine BOLD v. 2 [35].

Ten autosomal microsatellite loci mapped on chromosome-3 of *An. gambiae* were genotyped [36, 37] (see Additional file 1). Microsatellites located on chromosomes-X and -2 were not used to prevent bias due to gender (as larvae were not sexed) and selective pressures associated with paracentric inversions known to be frequent on chromosome-2 [38]. However, two of these microsatellites (AG3H119 and AG3H555) are located within inversion 3Ra (between divisions 31A and 34D), which is a polymorphic inversion in *An. arabiensis* [39]. Amplification was performed by PCR using forward primers labelled with 5’ fluorescent dyes (FAM, NED or HEX, Applied Biosystems, Foster City CA, USA) as described previously [40]. Amplified fragments were separated by capillary electrophoresis in an automatic sequencer ABI 3730 (Applied Biosystems, Foster City CA, USA) at Yale University’s DNA Analysis Facility (New Haven, CT, USA). Fragment sizes were scored using the software GeneMarker v1.4 (SoftGenetics, State College, PA, USA).

### Data analysis

Ninety-five per cent confidence intervals of proportions based on the sample size were calculated with continuity correction as described by Newcombe [41] and implemented in VassarStats [42]. Comparisons between groups were made by Pearson’s Chi-square tests on contingency tables or Fisher’s exact tests in the case of low sample sizes.

Microsatellite genetic diversity per locus and sample was characterized by estimates of allele richness (*A*_*R*_) [43], Nei’s unbiased expected heterozygosity *H*_e_ [44] and inbreeding coefficient *F*_IS_ [45]. Calculations were performed in FSTAT v. 2.9.3.2 [46]. Significance of mean *F*_IS_ values was assessed by randomization tests also available in FSTAT (1,400 replicates). Comparisons among samples of mean over-loci estimates of *A*_*R*_, *H*_e_ , and *F*_IS_ were performed by calculating bootstrapped 95% confidence intervals (1,000 replicates) using the Bootstrap Plot for Central Tendency v. 1.0.13 [47]. Genotypic frequencies were tested against Hardy-Weinberg Equilibrium (HWE) by exact probability tests performed in GENEPOP v. 4.1 [48]. The same software was used to perform exact tests of genotypic linkage disequilibrium between pairs of loci in each sample. Presence of null alleles at each locus and sample was assessed using the software MICRO-CHECKER v.2.2.3 [49].

Microsatellite loci isolated from one species (focal species) may be less variable in closely related species, due to ascertainment bias during the selection of microsatellite loci, in which loci with the longest tracts of pure repeats are usually favoured in order to ensure polymorphism [50, 51]. Since ascertainment bias is expected to increase with microsatellite length, interspecies diversity differences should be higher for loci with longer repeat tracts in *An. gambiae* and a stronger correlation between genetic diversity and repeat tract length is expected for the focal species. To test this hypothesis, estimates of allele richness were plotted against the length of the repeat tract (in repeat units) of the microsatellites described in the original clones [36, 37]. Analyses were conducted with and without loci AG3H93 and 45C1 because microsatellites with interrupted repeat motifs tend to be less variable than loci with pure repeat motifs [50]. Spearman rank correlation coefficients were estimated in SPSS v.20.0 [52] for *An. arabiensis* (larvae and adults pooled) and a pooled sample of *An. coluzzii* and *An. gambiae* (adults and larvae) representing the focal species (the isolation of the microsatellites predates the description of molecular form divergence). Fisher’s r-to-z tests, available in VassarStats, were used to assess the significance of the difference between two correlation coefficients.

In order to assess the degree of relatedness among individuals within samples, estimates of Queller and Goodnight [53] and Lynch and Ritland [54] relatedness coefficients were calculated using GENALEX v.6.5 [55]. Significance of the mean estimates for each sample was assessed by permutation tests (1,000 replicates) and bootstrapped 95% confidence intervals (1,000 replicates). In addition, the maximum-likelihood method implemented in ML-RELATE [56] was used to determine proportions of related individuals within samples. For each pair of individuals, log-likelihood estimates are calculated for four pedigree classes: unrelated, parent-offspring, full-siblings, and half-siblings. In loci displaying presence of null alleles, relatedness calculations were adjusted by including maximum likelihood estimates of the frequency of the putative null allele [57]. Individual pairs classified as relatives (i.e., PO, FS or HS) were summed in order to calculate the proportion of related individual pairs in each sample.

Single-sample estimates of current effective population size were calculated by the bias-corrected linkage disequilibrium method described in Waples and Do [58], as implemented in NeEstimator v.2 [59]. Because rare alleles may bias linkage disequilibrium *N*_e_ estimates, alleles with frequency below 0.05 at each locus were removed from the analysis.

Heterozygosity tests to detect deviations from mutation-drift equilibrium (MDE) were performed using BOTTLENECK v1.2.02 [60]. In these tests, two estimates of heterozygosity are compared: one based on allele frequencies assuming HWE (*H*_e_) and the other based on the number of alleles and sample size assuming MDE (*H*_eq_). At MDE, both estimates should be similar at the majority of the loci analysed (i.e,. *H*_e_ = *H*_eq_). In case of a population bottleneck, allelic diversity will decrease faster than heterozygosity (*i.e. H*_e_ > *H*_eq_), while the opposite (i.e., *H*_e_ < *H*_eq_) is an indicator of a population expansion. Estimates of expected heterozygosity under MDE were performed using the stepwise mutation model (SMM) and a two-phased model (TPM) with 10 to 20% indels larger than the repeat unit.

Whenever multiple tests were performed, the sequential Bonferroni procedure was applied to adjust the nominal significance level (α = 0.05) [61].

## Results

A total of 305 anopheline larvae (95 from the rice field and 210 from the temporary puddles) and 339 adult females (294 from CDC light traps and 45 from indoor-resting collections) were collected in the 2010 mosquito survey. While all 210 larvae collected in the temporary puddles were successfully amplified for both IGS and SINE, no amplified product was obtained for both markers in 79 (83.2%) out of the 95 specimens collected in the rice field. A subsample of 30 negative larvae was sequenced for the mtDNA COI fragment to perform species identification in BOLD (see Additional file 2). Of these, 28 individuals were identified as *Anopheles coustani* (99.3-100.0% similarity), one as *Uranotaenia balfouri* (99.7% similarity) and one specimen was assigned to an undetermined *Anopheles* species (*Anopheles* MBI-14, 99.4% similarity). An NCBI BLAST of the mtDNA COI sequence for this specimen gave a 91.0% similarity with *Anopheles funestus*. In the adult sample, one (2.2%) indoor-resting and four (1.4%) CDC light trap collected specimens had a readable genotype only for either the IGS or the SINE markers. These specimens were removed from subsequent analyses. Molecular identification by both IGS and SINE was thus achieved for a total of 560 specimens (226 larvae and 334 adults) from the 2010 collection. Additionally, a total of 553 indoor-resting females from collections undertaken between 1993 and 2007 were identified to species by both markers.

*Anopheles gambiae* and *An. arabiensis* were the predominant species sampled in the 2010 collection (Table 1). *Anopheles arabiensis* comprised 54.0% of the overall larval sample but only 3.8 and 22.7% of adults collected by CDC light traps and indoor-resting capture, respectively. *Anopheles coluzzii* larvae were collected only in temporary puddles, reaching a frequency of 10.5% which was *ca*. two-fold greater than those recorded in the adult samples. The frequency of this species was below 5.0% in the adult samples. *Anopheles melas* was identified only in adult samples, with an overall frequency of 2.7%.

**Table 1:** **Species distribution (in percentage) according to mosquito life-stage and collection site or method**

		***N***	***Anopheles arabiensis***	***Anopheles coluzzii***	***Anopheles gambiae***	Admixed	***Anopheles melas***
Larvae	Puddles	210	52.9	10.5	24.8	11.9	0.0
			[45.9-59.7]	[6.8-15.6]	[19.2-31.3]	[8.0-17.3]	[0.0-2.2]
	Rice field	16	68.8	0.0	6.3	25.0	0.0
			[41.5-87.9]	[0.0-24.1]	[0.3-32-3]	[8.3-52.6]	[0.0-24.1]
	Subtotal	226	54.0	9.7	23.5	12.8	0.0
			[47.3-60.6]	[6.3-14.6]	[18.2-29.6]	[8.9-18.1]	[0.0-2.1]
Adults	LT	290	3.8	4.8	43.8	45.2	2.4
			[2.0-6.9]	[2.8-8.2]	[38.0-49.7]	[39.4-51.1]	[1.1-5.1]
	IR	44	22.7	4.5	43.2	25.0	4.5
			[12.0-38.2]	[0.8-16.7]	[28.7-58.9]	[13.7-40.7]	[0.8-16.7]
	Subtotal	334	6.3	4.8	43.7	42.5	2.7
			[4.0-9.6]	[2.9-7.8]	[38.4-49.2]	[37.2-48.0]	[1.3-5.2]
Total		560	25.5	6.8	35.5	30.5	1.6
			[22.0-29.4]	[4.9-9.3]	[31.6-39.7]	[26.8-34.6]	[0.8-3.1]

*N*: sample size; LT: CDC light trap collection; IR: indoor resting collection. In square brackets: 95% confidence intervals.

The relative proportions of *An. coluzzii*, *An. gambiae* and admixed individuals (i.e., excluding *An. arabiensis* and *An. melas*) were significantly different when larval and adult samples were compared (χ^2^ = 26.70; d.f. 2; *P* <0.001; Additional file 3). *Anopheles gambiae* prevailed over *An. coluzzii* in both larval (51.0%) and adult (48.0%) samples. The relative proportion of *An. coluzzii* decreased from 22.1% in larvae to 5.3% in adults (χ^2^ = 23.17; d.f. 1; *P* <0.001), while relative proportion of admixed individuals nearly doubled from larvae (27.9%) to adults (46.7%) (χ^2^ = 11.28; d.f. 1; *P* <0.001).

**Figure 2:**
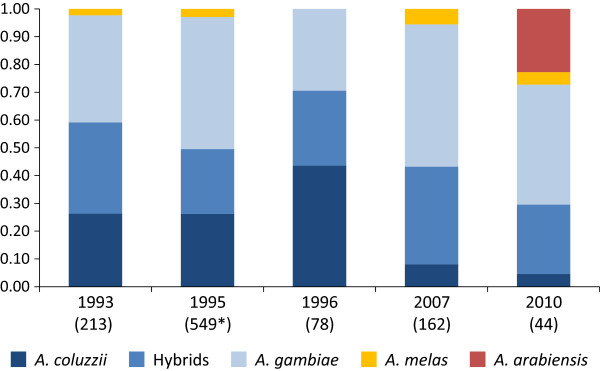
**Temporal variation of species composition of the** ***Anopheles gambiae*** **complex in indoor-** **resting collections Y** **-** **axis** **:** **proportion of each species** **;** **X-** **axis:** **years of collection and sample sizes in brackets.** *The 1995 sample includes data from Palsson *et al*. [21] from which a subsample of 100 individuals was identified by both IGS and SINE.

The detection of *An. arabiensis* in the study area was unexpected, as this species was not identified in previous indoor-resting samples collected since 1993 (Figure 2). Given the sample sizes of each year, the upper 95% confidence levels (UCL) for *An. arabiensis* to be present but not sampled in these previous years (1993: UCL = 2.2%, *N* = 213; 1995: UCL = 0.9%, *N* = 549; 1996: UCL = 5.9%, *N* = 78; 2007: UCL = 2.9%, *N* = 162) do not overlap with the confidence interval obtained for the proportion of *An. arabiensis* recorded in 2010 (95%CI: 12.0-38.2%, *N* = 44). Figure 2 also shows an apparent increase of the relative frequency of *An. gambiae* with a concomitant decrease of *An. coluzzii*. With the exception of the sample of 1996 (43.6%), the proportion of *An. coluzzii* in indoor resting samples went from 26.3% in 1993 to 4.5% in 2010.

### Microsatellite-based analyses

A total of 265 specimens collected in 2010 were genotyped for ten chromosome-3 microsatellite loci. Since the objective of this analysis was to perform interspecific comparisons, admixed individuals (as well as the few *An. coluzzii* and *An. melas* adults available) were excluded from the analysis.

Significant departures from HWE expectations were detected in five out of 50 tests performed, in the samples of *An. arabiensis* larvae (AGH758), *An. gambiae* adults (AG3H93) and *An. gambiae* larvae (AG3H119, AG3H242 and AG3H249) (Additional file 1). These departures were associated with positive *F*_IS_ values suggesting heterozygote deficits. Analysis performed by MICRO-CHECKER detected the presence of null alleles in three out of the five loci displaying heterozygote deficits. Loci AG3H119 and AG3H555, located within polymorphic inversion 3Ra in *An. arabiensis*, did not show any departures from HWE expectations in this species. These loci were not outliers for estimates of *A*_*R*_, *H*_*e*_ or *F*_IS_, suggesting that microsatellite polymorphism does not seem to have been affected by the possible presence of 3Ra inversion polymorphism in the *An. arabiensis* population sampled. There was no particular association between pairs of loci in any of the samples. Of the seven significant pairwise tests of linkage disequilibrium (out of 225 performed), four were detected in the sample of *An. arabiensis* larvae, one in *An. coluzzii* larvae and two in *An. gambiae* larvae. The low number of loci with heterozygote deficits and of linkage disequilibrium tests is consistent with each sample representing a single panmictic gene pool.

Mean estimates of genetic diversity were similar between larval and adult samples within the same species (Table 2). *Anopheles arabiensis* was less genetically diverse than *An. coluzzii* or *An. gambiae*. Mean allele richness for this species was around six alleles per locus whereas it varied between nine and ten in *An. coluzzii* and *An. gambiae*, respectively. Similarly, mean expected heterozygosity in *An. arabiensis* was below 0.600 while values above 0.800 were recorded for *An. coluzzii* and *An. gambiae*. These differences were significant judging from the non-overlapping bootstrapped 95% confidence intervals.

**Table 2:** **Mean estimates of genetic diversity and relatedness**

		***A***_R_	***H***_e_	***F***_IS_	***R***_LR_	***R***_QG_	***R***_ML_
*Anopheles arabiensis*	Adults	5.7	0.583	-0.054	**0.054**	**0.304**	17.9
	(*N* = 20)	[4.5-6.9]	[0.431-0.708]	[-0.132-0.017]	[0.042-0.063]	[0.279-0.327]	[12.9-24.3]
	Larvae	5.8	0.598	0.024	**0.049**	**0.268**	18.3
	(*N* = 122)	[4.7-6.8]	[0.455-0.706]	[-0.013-0.064]	[0.047-0.050]	[0.264-0.272]	[17.5-19.3]
*Anopheles coluzzii*	Larvae	8.8	0.812	0.017	**0.023**	0.027	6.5
	(*N* = 22)	[7.5-10.5]	[0.745-0.870]	[-0.052-0.086]	[0.017-0.030]	[0.009-0.047]	[3.8-10.7]
*Anopheles gambiae*	Adults	10.0	0.819	**0.082**	**0.019**	0.022	9.8
	(*N* = 48)	[8.1-11.9]	[0.767-0.864]	[0.055-0.113]	[0.016-0.021]	[0.013-0.031]	[8.1-11.7]
	Larvae	9.7	0.812	**0.079**	**0.015**	0.020	10.0
	(*N* = 53)	[7.9-11.7]	[0.756-0.863]	[0.022-0.130]	[0.013-0.019]	[0.012-0.028]	[8.5-11.8]

*N*: sample size; *A*_*R*_: allele richness; *H*_*e*_: expected heterozygosity; *F*_IS_: inbreeding coefficient; *R*_LR_: Lynch and Ritland [54] relatedness coefficient; *R*_QG_: Queller and Goodnight [53] relatedness coefficient; *R*_ML_: proportion (in percentage) of related pairs of individuals as determined by ML-RELATE. Significant estimates after correction of multiple tests for *F*_IS_, *R*_LR_ and *R*_QG_ are in bold. 95% confidence intervals are in square brackets.

Positive Spearman’s rho correlation coefficients between allele richness and repeat length of the original microsatellite clone were obtained for both *An. arabiensis* (rho = 0.648, *P* = 0.043) and *An. gambiae*/*An. coluzzii* (rho = 0.349, *P* = 0.323) (see Additional file 4). These coefficients were not statistically different (Fisher’s r-to-z test: z = 0.760, *P* = 0.447), and when only microsatellites with pure repeat motifs were analysed, correlation coefficients were near-identical (*An. arabiensis*: rho = 0.686, *P* = 0.060; *An. gambiae*/*An. coluzzii*: rho = 0.713, *P* = 0.047; Fisher’s r-to-z test: z = 0.080, *P* = 0.936).

There was no evidence for increased inbreeding in larval samples compared to the adult samples in both *An. arabiensis* and *An. gambiae* (Table 2). The latter species presented the highest and only significant mean *F*_IS_ estimates but these values were also practically identical between larvae and adults. Similarity between larval and adult samples was also evident in the values obtained for the estimators of relatedness in both species (Table 2; see Additional file 5). However, *An. arabiensis* exhibited the highest estimates for both relatedness coefficients and for the proportion of related individuals, when compared to the other two species. These differences were significant, judging from the non-overlapping confidence intervals, suggesting a higher degree of relatedness among *An. arabiensis* individuals in both larvae and adults (Table 2).

Mean estimates of effective population size varied between 32.8 in *An. arabiensis* adults and 192.4 in *An. gambiae* adults, with the latter estimate being the only with an unbounded 95% confidence interval (Table 3). However, all estimates had overlapping 95% confidence intervals indicative of no significant differences in *N*_e_ among samples. A significant departure from MDE was detected in both larval and adult samples of *An. arabiensis* (Table 3). This departure corresponded to a significant number of loci with an apparent deficit of heterozygotes when compared to expectations under MDE (i.e., *H*_e_ < *H*_eq_), an indicator of recent population expansion, and was consistent in all mutation models. A signal of population expansion was also detected in *An. gambiae* but only under the SMM and TPM 10% mutation models, in the case of the adult sample, and under the SMM in the case of the larval sample. There was no evidence of departure from MDE in the *An. coluzzii* sample.

**Table 3:** **Estimates of effective population size and heterozygosity tests**

		***N***_e_	Heterozygosity tests		
			SMM	TPM (10%)	TPM (20%)
*An. arabiensis*	Adults	32.8	9	9	9
		[15.1-253.0]	<**0.001**	**0.002**	**0.002**
	Larvae	88.3	10	10	10
		[54.7-166.4]	<**0.001**	<**0.001**	<**0.001**
*An. coluzzii*	Larvae	38.5	5	5	2
		[23.2-86.7]	0.278	0.615	0.884
*An. gambiae*	Adults	192.4	9	8	8
		[84.3-∞]	<**0.001**	**0.003**	0.007
	Larvae	114.8	9	7	6
		[61.7-415.3]	**0.001**	0.012	0.097

*N*_e:_ linkage disequilibrium-based estimate of effective population size and Jackknife 95% confidence interval in square brackets. Heterozygosity tests: upper values are the number of loci (out of 10) in which *H*_e_ < *H*_eq_; lower values are the *P*-*value* for the corresponding one-tailed Wilcoxon test. SMM: stepwise mutation model; TPM (10%): two-phased model with 10% of indels greater than one repeat; TPM (20%): two-phased model with 20% of indels greater than one repeat.

## Discussion

This study reports for the first time the occurrence of the major malaria vector *An. arabiensis* in Bissau, the capital city of Guinea Bissau. This was an unexpected finding since adult mosquito surveys carried out in the same area (Antula) and with the same collection method (indoor resting) did not sample this species [16, 18, 19, 21, 22] (Figure 2). In fact, *An. arabiensis* was reported over 30 years ago in Guinea Bissau, but its distribution appeared to be limited to the northeast inland region of the country, characterized by a drier savannah ecosystem [16].

The new occurrence of *An. arabiensis* in the humid coastal region of Guinea Bissau could have resulted from a very recent (after 2007) introduction by sporadic migration of a single or few females from north-eastern inland populations. If this was the case, then the expectation would be for an *An. arabiensis* population with low genetic diversity, small effective population size and with a signal of population contraction as a consequence of a recent founder event. Allele richness and expected heterozygosity estimated for both larval and adult *An. arabiensis* samples were indeed lower than those of *An. coluzzii* and *An. gambiae. Anopheles arabiensis* also presented a higher degree of relatedness among individuals. However, estimates of current *N*_e_ obtained for *An. arabiensis* were comparable to those of *An. gambiae* and *An. coluzzii*. While these estimates may be affected by the relatively low number of microsatellites analysed and small sample sizes, *An. arabiensis* does not seem to have a dramatically reduced *N*_e_ when compared to its sibling species, and no signal of population contraction was detected by heterozygosity tests. Instead, an apparent heterozygote deficit, relative to equilibrium expectations, was detected consistently in both larval and adult *An. arabiensis* samples, suggesting population expansion. This result agrees with the apparent increase of the relative frequency of this species in 2010, which reached 22.7% (12.0-38.2%) in indoor-resting collections, a value far greater than the maximum upper level confidence limit estimated for previous collections (5.9%, in 1996). Therefore, although the possibility of a new colonization in Bissau cannot be excluded, a more likely explanation is that of an expanding *An. arabiensis* population that has been resident for some time and that other causes may underlie the reduced levels of diversity.

The unavailability of microsatellite loci isolated specifically from *An. arabiensis* precluded performing reciprocal tests [50] so that ascertainment bias cannot be fully rejected. However, correlation coefficients between allele diversity and microsatellite repeat tract length were similar for both *An. arabiensis* and *An. gambiae* + *An. coluzzii*, indicating that allele diversity was consistently lower in *An. arabiensis* irrespective of the originally cloned repeat tract length (which varied between six and 21 repeats). This similarity in the diversity *vs* tract length relationship across species argues against ascertainment bias as an explanation for reduced genetic diversity of the microsatellite loci in *An. arabiensis*.

Another hypothesis is that the low genetic diversity found in *An. arabiensis* reflects a small but now-expanding exophagic and exophilic population living at the edge of the species distribution. This population could have remained undetected in collections carried out before 2010 due not only to its low abundance, but also to inadequate sampling methodology. The difference in the relative frequency of *An. arabiensis* between larval and adult samples supports this hypothesis. In 2010, *An. arabiensis* was the most frequent species in larval collections (exceeding 50%) but this was not apparent in the indoor adult samples, particularly in CDC light trap collections, where its frequency was lower than 4%. This suggests the presence of a markedly exophagic and exophilic *An. arabiensis* in Antula, difficult to catch using CDC light trap and resting collections performed indoors, which sample preferentially endophagic and endophilic fractions of a mosquito population. Bio-ecological studies point to a greater behavioural plasticity of *An. arabiensis* when compared to *An. coluzzii* and *An. gambiae* (see [1] for a review). Populations of this species frequently display higher degrees of exophagy and exophily, which is often associated with a higher propensity to feed on non-human hosts. Moreover, the introduction of insecticide-treated bed nets in the area [23, 24] may also have contributed to the selection of outdoor behaviours in this species. Additional bio-ecological studies, involving outdoor collections and determination of host-preferences and gonotrophic state, will be required to confirm the extent of these behaviours in the *An. arabiensis* population of Antula. Such studies should also involve sampling in different collection sites in order to clarify if the reduced diversity is associated with the edge of the species distribution and the possible causes of the apparent population expansion detected in the 2010 sample.

There is also a possibility of the high proportion of *An. arabiensis* found in larvae having resulted from sampling a large number of siblings from a single or a few ovipositions, due to the relatively low number of larval habitats surveyed. However, this is not supported by estimates of genetic relatedness, which, although higher in *An. arabiensis*, were similar between adult and larval samples of this species as well as of *An. gambiae*, suggesting that larval and adult collections were equally representative of the population for both species. The proportion of related individuals determined in this study was lower than those reported for *An. gambiae* larvae from Kenya [62] and for adult samples from three African countries [63], but these studies have used different microsatellites and relatedness estimators. Furthermore, methods to estimate genetic relatedness require a large number of polymorphic loci (*ca*. 30-40) to fully discriminate individual pairs according to pedigree classes [56, 64]. This was evident in the present analysis when parent-offspring relations at frequency between 0.1 and 2.5% (see Additional file 5) were identified in larval samples, which is a biologically unsound result. However, even when based on a few loci these approaches may still be useful when the primary goal is to compare the average relatedness within groups [64], as was the case of the present study.

The increasing relative frequencies of *An. gambiae* since 1993 and the high levels of genetic diversity with a signal of population expansion observed in 2010 suggest that this species is also expanding in the study area. This may be due to a higher fitness derived from integration of *An. coluzzii* genetic variants following asymmetric genetic introgression from *An. coluzzii* to *An. gambiae* in this secondary contact zone as previously shown [5, 14] and here confirmed by the increased proportion of admixed individuals in the adult collections. Additional surveys targeting the dry season and involving genetic analyses are required to confirm whether the apparent dominance of *An. gambiae* and *An. arabiensis* over *An. coluzzii* in the study site is a stable situation or if it corresponds to a seasonal fluctuation. In fact, the frequency of *An. coluzzii* was higher than *An. gambiae* in the only adult sample collected at the onset of the dry season (i.e., November 1996), contrasting with the other samples that were collected in the rainy season. This is possibly due to *An. coluzzii* higher capacity to explore more permanent larval habitats [11, 13].

The initial goal of assessing spatial segregation at the larval stage between *An. coluzzii* and *An. gambiae* in a setting of high hybridization was hampered by the low numbers obtained for these species in the collections made in the permanent larval habitat. Of the 95 larvae collected in the rice field, only 16 belonged to the *An. gambiae* complex and 11 were identified as *An. arabiensis*. Morphological identification of mosquito species at the larval stage sometimes implies mounting the biological material in slides for microscopic observation of diagnostic structures, which can be difficult under certain field conditions. In this context, DNA barcoding may be an effective alternative. This approach reliably identified a subsample of the larvae, revealing a large predominance of *An. coustani* in the rice field larval collection made. Members of this species complex are considered secondary malaria vectors due to a high degree of zoophily and exophagy. However, a few studies suggest a role in malaria transmission, which may justify further attention to this complex in future malaria vector surveys in area [65, 66].

## Conclusions

The finding of an *An. arabiensis* population in Antula, apparently displaying exophilic behaviour, has important implications for the epidemiology and control of malaria. Previous malaria control programmes, such as the Garki Project in the 1970s, have demonstrated that outdoor feeding and resting mosquito populations can undermine vector control [67, 68] which is still based mainly on indoor measures. This is of particular relevance for malaria control efforts in Guinea Bissau given that free distribution of insecticide-treated nets has been the mainstay of vector control in the country since 2005 [23, 24]. Finally, these results highlight the importance of complementing indoor mosquito sampling with alternative methods targeting outdoor adult mosquitoes and immature stages, for a more representative sampling of the mosquito biodiversity in a given region.

## Electronic supplementary material

Additional file 1: **Estimates of genetic diversity per microsatellite locus.** (DOCX 22 KB)

Additional file 2: **Species identification by DNA barcoding using BOLD database.** (DOCX 18 KB)

Additional file 3: **Number and proportion (percentages in parenthesis) of** ***Anopheles coluzzii*** **,** ***Anopheles gambiae*** **and admixed individuals in larval and adult samples.** (DOCX 14 KB)

Additional file 4: **Correlation between allele richness and length of the original microsatellite clone isolated from** ***Anopheles gambiae.*** (DOCX 44 KB)

Additional file 5: **Proportion (in percentage) of individuals assigned to each pedigree class by ML-RELATE [** [56]**].** (DOCX 16 KB)
