# Supplementary material for: First report of an exophilic Anopheles arabiensis population in Bissau City, Guinea-Bissau: recent introduction or sampling bias?
Source: Malar J. 2014 Nov 4;13:423. doi: 10.1186/1475-2875-13-423 (PMC4240859; doi:10.1186/1475-2875-13-423)
Supplement: Supplementary file 1 — Additional file 1: Estimates of genetic diversity per microsatellite locus. (DOCX 22 KB) [file 12936_2014_3589_MOESM1_ESM.docx]

**Additional file 1. Estimates of genetic diversity per microsatellite locus.**

|  |  | *Anopheles arabiensis* | | *Anopheles coluzzii* | *Anopheles gambiae* | |
| --- | --- | --- | --- | --- | --- | --- |
|  |  | Adults | Larvae | Larvae | Adults | Larvae |
|  |  | (*N*=20) | (*N*=122) | (*N*=22) | (*N*=48) | (*N*=53) |
| AG3H93 | *A_R_* | 6.0 | 7.3 | 8.8 | 11.0 | 11.4 |
| GT_4+7_; 29A | *H_e_* | 0.626 | 0.711* | 0.859 | **0.837*** | 0.848 |
|  | *F_IS_* | 0.112 | 0.123 | 0.048 | 0.187 | 0.021 |
| AG3H128 | *A_R_* | 8.7 | 6.9 | 13.7 | 16.8 | 16.5 |
| GT_21_; 29C | *H_e_* | 0.741 | 0.683 | 0.915 | 0.938 | 0.938 |
|  | *F_IS_* | 0.055 | 0.064 | 0.056 | 0.067 | 0.095 |
| AG3H59 | *A_R_* | 5.8 | 6.2 | 6.5 | 7.8 | 8.1 |
| GT_9_; 29D | *H_e_* | 0.640 | 0.713 | 0.754 | 0.782 | 0.799 |
|  | *F_IS_* | 0.014 | -0.035 | -0.145 | 0.067 | 0.008 |
| AG3H249 | *A_R_* | 6.7 | 8.9 | 9.7 | 10.9 | 10.9 |
| GT_15_; 30C | *H_e_* | 0.695 | 0.767 | 0.880 | 0.860 | **0.876** |
|  | *F_IS_* | -0.080 | 0.027 | 0.134 | 0.007 | 0.117 |
| AG3H119 | *A_R_* | 5.9 | 6.0 | 11.9 | 11.4 | 11.3 |
| GT_6_; 31B | *H_e_* | 0.775 | 0.732 | 0.896* | 0.863 | **0.892*** |
|  | *F_IS_* | -0.161 | -0.008 | 0.239 | 0.059 | 0.133 |
| AG3H555 | *A_R_* | 2.9 | 3.0 | 6.9 | 7.7 | 7.5 |
| GT_8_; 32C | *H_e_* | 0.188 | 0.235 | 0.831 | 0.819 | 0.812 |
|  | *F_IS_* | -0.063 | -0.030 | 0.016 | 0.039 | 0.141 |
| AG3H577 | *A_R_* | 6.9 | 6.6 | 7.6 | 10.8 | 8.4 |
| GT_16_; 42A | *H_e_* | 0.772 | 0.742 | 0.635 | 0.780 | 0.673 |
|  | *F_IS_* | 0.045 | -0.039 | 0.070 | 0.127 | 0.019 |
| AG3H758 | *A_R_* | 4.0 | 3.9 | 10.4 | 11.8 | 10.2 |
| GT_11_; 43A | *H_e_* | 0.570 | **0.558** | 0.881 | 0.898 | 0.856* |
|  | *F_IS_* | -0.141 | 0.105 | -0.032 | 0.080 | 0.175 |
| AG3H242 | *A_R_* | 2.8 | 2.8 | 5.9 | 5.8 | 6.2 |
| GT_8_; 45C | *H_e_* | 0.099 | 0.190 | 0.641 | 0.634 | **0.638*** |
|  | *F_IS_* | -0.013 | 0.095 | -0.064 | 0.113 | 0.202 |
| 45C1 | *A_R_* | 7.8 | 5.9 | 6.8 | 5.6 | 6.2 |
| TG_4+7+4_; 45C | *H_e_* | 0.728 | 0.647 | 0.826 | 0.778 | 0.788 |
|  | *F_IS_* | -0.306 | -0.064 | -0.156 | 0.078 | -0.126 |

Legend: Information on the repeat motif and size of the original sequenced clone and chromosomal location for each microsatellite is given below the locus name. Chromosomal locations were based on Zheng *et al.* [36] and confirmed in VectorBase (www.vectorbase.org). *A_R_*: allele richness; *H_e_*: expected heterozygosity; *F_IS_*: inbreeding coefficient. In bold: significant HWE tests after sequential Bonferroni correction. * Presence of null alleles.
