# Supplementary material for: First report of an exophilic Anopheles arabiensis population in Bissau City, Guinea-Bissau: recent introduction or sampling bias?
Source: Malar J. 2014 Nov 4;13:423. doi: 10.1186/1475-2875-13-423 (PMC4240859; doi:10.1186/1475-2875-13-423)
Supplement: Supplementary file 2 — Additional file 2: Species identification by DNA barcoding using BOLD database. (DOCX 18 KB) [file 12936_2014_3589_MOESM2_ESM.docx]

**Additional file 2. Species identification by DNA barcoding using BOLD database.**

| **ID nº** | **GENBANK** | **Species (BOLD)** | **% similarity** |
| --- | --- | --- | --- |
| SeqANBI01 | KM097001 | *Anopheles coustani* | 99.8 |
| SeqANBI02 | KM097002 | *Anopheles coustani* | 99.3 |
| SeqANBI03 | KM097003 | *Anopheles coustani* | 99.5 |
| SeqANBI04 | KM097004 | *Anopheles coustani* | 99.4 |
| SeqANBI06 | KM097005 | *Anopheles coustani* | 99.4 |
| SeqANBI08 | KM097006 | *Anopheles coustani* | 99.4 |
| SeqANBI09 | KM097007 | *Anopheles coustani* | 99.8 |
| SeqANBI11 | KM097029 | *Anopheles MBI-14** | 99.4 |
| SeqANBI12 | KM097008 | *Anopheles coustani* | 99.4 |
| SeqANBI13 | KM097030 | *Uranotaenia balfouri* | 99.7 |
| SeqANBI14 | KM097009 | *Anopheles coustani* | 100.0 |
| SeqANBI15 | KM097010 | *Anopheles coustani* | 99.7 |
| SeqANBI16 | KM097011 | *Anopheles coustani* | 99.4 |
| SeqANBI17 | KM097012 | *Anopheles coustani* | 99.8 |
| SeqANBI18 | KM097013 | *Anopheles coustani* | 99.4 |
| SeqANBI19 | KM097014 | *Anopheles coustani* | 99.4 |
| SeqANBI20 | KM097015 | *Anopheles coustani* | 99.3 |
| SeqANBI21 | KM097016 | *Anopheles coustani* | 99.4 |
| SeqANBI22 | KM097017 | *Anopheles coustani* | 99.8 |
| SeqANBI23 | KM097018 | *Anopheles coustani* | 99.4 |
| SeqANBI24 | KM097019 | *Anopheles coustani* | 99.5 |
| SeqANBI25 | KM097020 | *Anopheles coustani* | 99.3 |
| SeqANBI26 | KM097021 | *Anopheles coustani* | 99.8 |
| SeqANBI27 | KM097022 | *Anopheles coustani* | 100.0 |
| SeqANBI28 | KM097023 | *Anopheles coustani* | 100.0 |
| SeqANBI29 | KM097024 | *Anopheles coustani* | 99.4 |
| SeqANBI30 | KM097025 | *Anopheles coustani* | 99.4 |
| SeqANBI31 | KM097026 | *Anopheles coustani* | 99.8 |
| SeqANBI32 | KM097027 | *Anopheles coustani* | 99.7 |
| SeqANBI33 | KM097028 | *Anopheles coustani* | 99.7 |

Legend: ID nº: specimen identification code; GENEBANK: accession number at NCBI Genebank; % similarity: sequence similarity with reference species in BOLD database. * One undetermined species (APL11) gave 91% similarity with *A. funestus* sequences in a BLAST search at NCBI GenBank.
