# Supplementary material for: First report of an exophilic Anopheles arabiensis population in Bissau City, Guinea-Bissau: recent introduction or sampling bias?
Source: Malar J. 2014 Nov 4;13:423. doi: 10.1186/1475-2875-13-423 (PMC4240859; doi:10.1186/1475-2875-13-423)
Supplement: Supplementary file 3 — Additional file 3: Number and proportion (percentages in parenthesis) of Anopheles coluzzii , Anopheles gambiae and admixed individuals in larval and adult samples. (DOCX 14 KB) [file 12936_2014_3589_MOESM3_ESM.docx]

**Additional file 3. Number and proportion (percentages in parenthesis) of *A. coluzzii*, *A. gambiae* and admixed individuals in larval and adult samples.**

|  | *N* | *Anopheles coluzzii* | *Anopheles gambiae* | Admixed |
| --- | --- | --- | --- | --- |
| Larvae | 104 | 22  (21.2) | 53  (51.0) | 29  (27.9) |
| Adults | 304 | 16  (5.3) | 146  (48.0) | 142  (46.7) |
