# Supplementary material for: First report of an exophilic Anopheles arabiensis population in Bissau City, Guinea-Bissau: recent introduction or sampling bias?
Source: Malar J. 2014 Nov 4;13:423. doi: 10.1186/1475-2875-13-423 (PMC4240859; doi:10.1186/1475-2875-13-423)
Supplement: Supplementary file 4 — Additional file 4: Correlation between allele richness and length of the original microsatellite clone isolated from Anopheles gambiae. (DOCX 44 KB) [file 12936_2014_3589_MOESM4_ESM.docx]

**Additional file 4. Correlation between allele richness and length of the original microsatellite clone isolated from *A. gambiae.***

Legend: X-axis: length of the microsatellite clone in number of repeats [36,37]; Y-axis: allele richness; Red squares: *A. gambiae*; Blue diamonds: *A. arabiensis*. A: all loci; B: only loci with pure repeat tracts (i.e. excluding AG3H93 and 45C1).
