# Supplementary material for: First report of an exophilic Anopheles arabiensis population in Bissau City, Guinea-Bissau: recent introduction or sampling bias?
Source: Malar J. 2014 Nov 4;13:423. doi: 10.1186/1475-2875-13-423 (PMC4240859; doi:10.1186/1475-2875-13-423)
Supplement: Supplementary file 5 — Additional file 5: Proportion (in percentage) of individuals assigned to each pedigree class by ML-RELATE [ [56]]. (DOCX 16 KB) [file 12936_2014_3589_MOESM5_ESM.docx]

**Additional file 5. Proportion (in percentage) of individuals assigned to each pedigree class by ML-RELATE [56]**

|  | *An. arabiensis* | | *An. coluzzii* | *An. gambiae* | |
| --- | --- | --- | --- | --- | --- |
|  | Adults | Larvae | Larvae | Adults | Larvae |
|  | (190) | (7381) | (231) | (1128) | (1378) |
| Full-sibling | 1.6 | 2.0 | 1.3 | 0.6 | 1.2 |
| Half-sibling | 12.6 | 13.8 | 4.8 | 8.9 | 8.7 |
| Parent/Offspring | 3.7 | 2.5 | 0.4 | 0.3 | 0.1 |
| Unrelated | 82.1 | 81.7 | 93.5 | 90.2 | 90.0 |

In parenthesis: number of individual pairs tested.
